# Cardiac looping may be driven by compressive loads resulting from unequal growth of the heart and pericardial cavity. Observations on a physical simulation model

**DOI:** 10.3389/fphys.2014.00112

**Published:** 2014-04-04

**Authors:** Meriç Bayraktar, Jörg Männer

**Affiliations:** Group Cardio-Embryology, Institute for Anatomy and Embryology, UMG, Georg-August-University of GöttingenGöttingen, Germany

**Keywords:** heart looping, mechanics, growth-induced buckling, simulation model, chirality

## Abstract

The transformation of the straight embryonic heart tube into a helically wound loop is named *cardiac looping*. Such looping is regarded as an essential process in cardiac morphogenesis since it brings the building blocks of the developing heart into an approximation of their definitive topographical relationships. During the past two decades, a large number of genes have been identified which play important roles in cardiac looping. However, how genetic information is physically translated into the dynamic form changes of the looping heart is still poorly understood. The oldest hypothesis of cardiac looping mechanics attributes the form changes of the heart loop (ventral bending → simple helical coiling → complex helical coiling) to compressive loads resulting from growth differences between the heart and the pericardial cavity. In the present study, we have tested the physical plausibility of this hypothesis, which we call the *growth-induced buckling hypothesis*, for the first time. Using a physical simulation model, we show that growth-induced buckling of a straight elastic rod within the confined space of a hemispherical cavity can generate the same sequence of form changes as observed in the looping embryonic heart. Our simulation experiments have furthermore shown that, under bilaterally symmetric conditions, growth-induced buckling generates left- and right-handed helices (D-/L-loops) in a 1:1 ratio, while even subtle left- or rightward displacements of the caudal end of the elastic rod at the pre-buckling state are sufficient to direct the buckling process toward the generation of only D- or L-loops, respectively. Our data are discussed with respect to observations made in biological “models.” We conclude that compressive loads resulting from unequal growth of the heart and pericardial cavity play important roles in cardiac looping. Asymmetric positioning of the venous heart pole may direct these forces toward a biased generation of D- or L-loops.

## Introduction

The heart is the first organ to form and function in vertebrate embryos. It arises from the merging of bilaterally paired heart-forming fields in front of the developing foregut (van den Berg et al., 2009; Kelly, 2012). During the initial phase of the merging of the heart-forming fields, the embryonic heart is seen as a straight and bilaterally almost symmetric tube-like structure oriented along the ventral midline of the foregut. This “*tubular*” heart is relatively short and consists only of the embryonic left ventricle. It has a venous inlet at its caudal pole, with which it is connected to the developing veins, and an arterial outlet at its cranial pole, with which it is connected to the aortic sac. Its dorsal wall is fixed to the dorsal wall of the embryonic pericardial cavity via a mid-sagittal tissue bridge called the *dorsal mesocardium*. During subsequent merging of the heart-forming fields, the initially short heart tube becomes elongated by continuous addition of new building blocks to its venous and arterial poles. The elongation of the tubular heart is accompanied (i) by the disappearance of the dorsal mesocardium, so that the tubular heart now is fixed to the wall of the embryonic pericardial cavity only at its venous (caudal) and arterial (cranial) ends; and (ii) by striking changes in the spatial configuration of the developing heart, which becomes transformed from a straight into a helically coiled tube (for review see Männer, 2009).

The transformation of the initially straight embryonic heart tube into a helically coiled heart loop is named *cardiac looping*. Such looping is regarded as an essential process in the morphogenesis of embryonic vertebrate hearts since it brings the building blocks of the developing heart and the stems of the great blood vessels into an approximation of their definitive topographical relationships (Männer, 2009).

**Figure 1:**
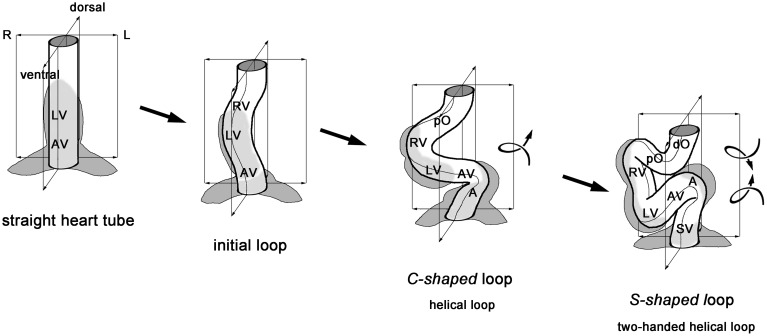
**This schematic drawing illustrates the sequence of idealized geometrical form changes characterizing the looping morphogenesis of the tubular heart of higher vertebrate embryos**. During the initial phase of looping, the straight tube starts bending along the mid-sagittal plane toward the ventral body wall. Rightward torsion deforms the bending tube into a helically coiled loop with a counter-clockwise winding (“C-shaped” loop). The loop finally acquires the complex helical configuration of a two-handed helix, which consists of a caudal limb with a counter-clockwise winding and a cranial limb with a clockwise winding (“S-shaped” loop). A, atrium; AV, atrio-ventricular canal; LV, embryonic left ventricle; dO, distal outflow tract; pO, proximal outflow tract; RV, embryonic right ventricle; SV, sinus venosus.

The sequence of the dynamic form changes of the looping heart tube of higher vertebrate embryos can be subdivided into three subsequent phases (Männer, 2000, 2009). During the first phase, the initially straight heart tube bends toward the ventral body wall and undergoes a torsion around its center axis so that it normally acquires the configuration of a helix with a single counter-clockwise (left-handed) winding (Figure 1). This heart loop looks like the Latin letter “*C*” when seen in a frontal two-dimensional projection (Männer, 2004) and, therefore, is frequently named the “*C-shaped*” heart loop. The convexity of the “*C*” normally points toward the right side of the embryo, which is the reason for naming the first phase of looping as the phase of *dextral-looping*. During the second phase, the heart loop acquires a complex helical shape, which is traditionally named the “*S-shaped*” heart loop since it resembles the Latin letter “*S*” when seen in a frontal two-dimensional projection. The complex spatial configuration of the “S-shaped” heart loop has recently been identified as a *helical perversion* (Männer, 2013). A helical perversion connects two helical segments of opposite handedness within the same helically coiled object. Such helical objects may, therefore, be named in a more descriptive way as *two-handed helices* (Pieranski et al., 2004). The spatial configuration of the so-called “S-shaped” heart loop of higher vertebrate embryos corresponds to a two-handed helix that is polarized along the cranio-caudal body axis and consists of a caudal limb with a left-handed helical winding and a cranial limb with a right-handed helical winding (Männer, 2013). These two limbs are connected with each other via a curved loop whose convexity normally points toward the right side of the embryo (Figure 1). Following the direction of blood flow, this heart loop configuration may be named *LR-handed helix*. The third and final phase of cardiac looping is characterized by form changes that may be summarized under the term “*untwisting*” of the heart loop (Männer, 2009).

Helices and polarized helical perversions are handed objects, which means that they can principally exist in two form variants—so-called *enantiomorphs*—, each of them being the mirror image of the other one (Männer, 2013). With respect to the embryonic vertebrate heart, this would mean that the so-called “C-shaped” and “S-shaped” heart loops principally could exist not only in the above-mentioned helical configurations (left-handed helix, LR-handed helix), but also in the form of mirror imaged enantiomorphs (right-handed helix, RL-handed helix). Interestingly, however, in all vertebrate species studied so far, only the above-mentioned enantiomorphs are normally being realized during embryonic development. Since the convexity of the ventricular portion of these loops points toward the right side of the embryo, these biologically normal enantiomorphs are generally named *D- (dextral) loops* while their biologically abnormal mirror images are classified as *L- (levo) loop*s.

Since the nineteenth century, the fascination for the phenomenon of cardiac looping has centered around two main questions: (i) what factor(s) drive the bending and coiling of the embryonic heart tube; and (ii) what factor(s) are responsible for the fact that, in all vertebrate species studied so far, only the so-called D-loop configuration is normally being realized during embryonic development?

During the past two decades, remarkable progress has been made in answering these two questions. A large number of genes and molecular signaling pathways have been identified which play important roles either (i) in the elongation, bending and coiling of the tubular heart or, (ii) in the determination of the handedness of heart loop configuration, suggesting that these two aspects of cardiac looping are controlled by distinct sets of factors (Männer, 2013). However, how genetic information and molecular signals are translated into mechanical forces driving the dynamic form changes of the looping embryonic heart is still an open question.

**Figure 2:**
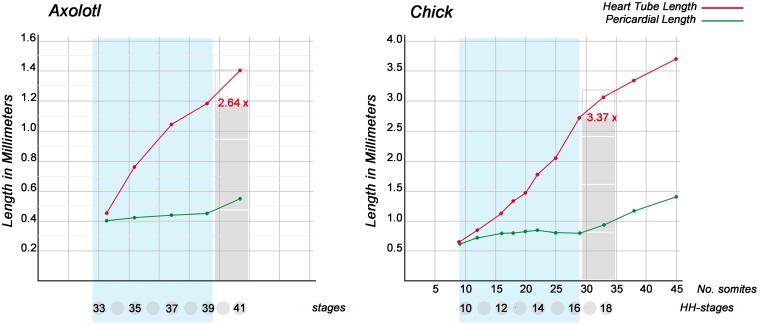
**These graphs show the dynamics of the elongation of looping embryonic heart tubes as compared with the cranio-caudal growth of pericardial cavities**. Graphs are based on data from Axoltol (Fransen and Lemanski, 1988) and chick embryos (Patten, 1922). Note that, during the phases of dextral-looping and early S-looping (marked in light blue), the cranio-caudal lengths of the pericardial cavities remain relatively constant while the heart tubes undergo considerable elongation. At the onset of looping, the straight heart tubes have the same lengths as the pericardial cavities. At the end of early S-looping, however, the heart loop of Axolotl embryos is 2.64 times longer, and the heart loop of chick embryos is 3.37 times longer, than the pericardial cavity.

The historically oldest concept of the biomechanics of cardiac looping regards the bending and helical coiling of the tubular embryonic heart as results of the continuous heart tube elongation within the confined space of the embryonic pericardial cavity (His, 1868; Goette, 1875; Tandler, 1912; Robertson, 1914; Patten, 1922; Davis, 1927; Bremer, 1928; Fransen and Lemanski, 1988). This view was largely based on the observation that, during the period of looping morphogenesis, the heart grows rapidly in length while the distance between its fixed venous and arterial ends remains relatively constant (Figure 2). If this hypothesis would be correct, the biological phenomenon of cardiac looping would correspond to a technical phenomenon which is well known to the drilling industry, namely the buckling of a long drill tube in a drill hole. A straight drill tube or drill string buckles within the narrow cylindrical space of a drill hole when it is subjected to an axial compressive load that exceeds a critical value, beyond which a straight shape is no longer stable and deforms into a sinusoidal or helical configuration (Tan and Forsman, 1995). In the case of the embryonic heart tube, it is likely that an axial compressive load is generated by the continuous addition of new heart field-derived material to its fixed arterial and venous ends. It, therefore, seems reasonable to name the historically oldest hypothesis of cardiac looping mechanics “*the growth-induced buckling hypothesis.*”

For several years, the growth-induced buckling hypothesis of cardiac looping mechanics was outside of the focus of contemporary research since it seemed that it had been disproven by the observation that embryonic heart tubes are able to undergo looping when explanted from the embryonic pericardial cavity and cultured *in vitro* without spatial restrictions (Ekman, 1925; Bacon, 1945; Butler, 1952; Manning and McLachlan, 1990). It has been found, however, that this “*in vitro looping*” is simpler than the normal “*in situ looping*” since it produces heart loops that are bend only in a single plane (planar looping) but lack a helical shape (spatial looping) (Flynn et al., 1991; Latacha et al., 2005; Rémond et al., 2006; Ramasubramanian et al., 2013). Contemporary hypotheses of cardiac looping mechanics, therefore, attribute the bending of the tubular embryonic heart primarily to forces intrinsic to its myocardial wall (Latacha et al., 2005; Taber, 2006; Taber et al., 2010) while its deformation into helical shapes is attributed primarily to factors outside of the heart (Taber, 2006; Taber et al., 2010; Männer, 2013). Recent experimental studies on chick embryos have shown that the ventral wall of the primary pericardial cavity exerts a compressive force against the bending heart tube without which the torsion of the looping heart does not occur properly (Voronov and Taber, 2002; Taber, 2006; Filas et al., 2007; Taber et al., 2010). This suggests that the size and geometry of the embryonic pericardial cavity are important extrinsic determinants of cardiac looping mechanics. It, furthermore, suggests that, if we want to gain full insight into the biomechanics of cardiac looping, we should no longer ignore the growth-induced buckling hypothesis.

In view of the above-mentioned recent experimental findings, we became interested in clarifying the possible role of growth-induced buckling in cardiac looping morphogenesis. As the first step on this way, we wanted to know whether the sequence of form changes characterizing cardiac looping (Figure 1) might correspond to the sequence of form changes characterizing the buckling of an elastic tube within a confined space. To our great surprise, we found that this question has never been addressed in embryological studies during the past 200 years. We, therefore, searched for an answer in the literature from engineering sciences dealing with the technical problem of drill string buckling (see above). Laboratory tests have shown that this process runs in two subsequent steps (Tan and Forsman, 1995). When subjected to an increasing axial load, a long drill string bends along a single plane (*planar* or *sinusoidal* buckling) until the middle of the bends contact the wall of the confining drill hole. The string then buckles in a three-dimensional fashion and finally acquires the configuration of a helix (*spatial* or *helical* buckling). Such form behavior would correspond to the form behavior characterizing the first phase of cardiac looping (*dextral*- or “*C*”*-looping*) and, therefore, would be in accord with the idea that growth-induced buckling may contribute to the generation of the helical shape of the “C-shaped” heart loop. Unfortunately, we could not find an answer to the question whether spatial buckling of a drill string may also generate the complex shape of a two-handed helix representing the idealized geometrical configuration of the so-called “S-shaped” heart loop. We, therefore, decided to construct a mechanical device (physical looping model) with which we wanted to test whether growth-induced buckling of a straight elastic rod (representing the embryonic heart tube) within the confined space of a hemispherical cavity (representing the embryonic pericardial cavity) can generate a sequence of form changes corresponding to cardiac looping (straight tube → sinusoidal loop → helical loop → two-handed helical loop).

**Figure 3:**
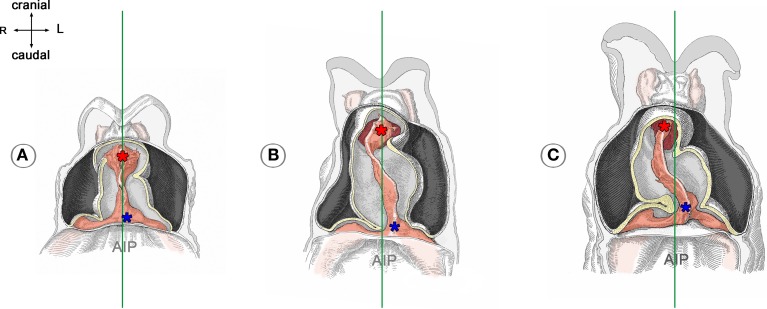
**These drawings depict the asymmetric positioning of the venous pole of human embryonic heart tubes before the onset (A) and during the initial steps of cardiac looping (B,C)**. Hearts are shown in frontal views within the opened pericardial cavities. The ventral myocardial walls have been removed to facilitate views on the endocardial tubes. **(A)** Before the onset of looping, the center axis of the venous heart pole (blue asterisk) is slightly displaced to the left of the mid-sagittal plane (marked by the green line). **(B,C)** The degree of leftward displacement of the venous pole increases during the initial steps of looping. Note also that the arterial pole undergoes rightward displacement. Drawings are based on Figures 18, 20, and 22 from Davis (1927). AIP, anterior intestinal portal.

If real or simulated cardiac looping would run under conditions of precise bilateral symmetry, the ratio of the resulting loop-enantiomorphs (D-/L-loop) is expected to be 1:1. It, therefore, has been speculated that the biologically normal ~100% preference for the D-loop enantiomorph may be caused by functional or structural asymmetries already present in the pre-looping embryo (Stalsberg, 1970). Studies on mouse, cat and human embryos (Schulte, 1916; De Vries and Saunders, 1962; Biben and Harvey, 1997) have shown that the venous end of the straight heart tube normally is displaced to the left shortly before overt looping (Figure 3), and it has been speculated that this pre-looping asymmetry may be a decisive event in determining the D-loop phenotype (von Baer, 1828; Schulte, 1916; Biben and Harvey, 1997). With respect to the growth-induced buckling hypothesis this speculation seems to make sense because leftward displacement of the fixed venous end of the embryonic heart tube is expected to result in a bilaterally asymmetric distribution of the axial compressive load along the heart tube, which, in turn, may direct the buckling process toward a statistically non-random generation of loop-enantiomorphs. We, therefore, decided to construct our above-mentioned physical model for cardiac looping simulations in a way that it facilitated testing of the growth-induced buckling behavior of a straight elastic rod under two different conditions: (i) bilateral symmetry, and (ii) bilateral asymmetry caused by left- or rightward displacement of its fixed caudal end before running the simulation. In the present study, we used this model to answer three questions: (i) Can growth-induced buckling of a straight elastic rod in a narrow hemispherical cavity generate the same sequence of form changes as observed in the looping embryonic heart? (ii) Can leftward displacement of the caudal end of the elastic rod direct the buckling process toward preferential generation of helical configurations corresponding to the cardiac D-loop enantiomorph; and can rightward displacement direct the buckling process toward preferential generation of helical configurations corresponding to the cardiac L-loop enantiomorph? (iii) Can even subtle left- or right-ward displacement of the caudal end of the elastic rod direct the buckling process toward a 100% generation of only one of two possible loop-enantiomorphs?

## Materials and methods

**Figure 4:**
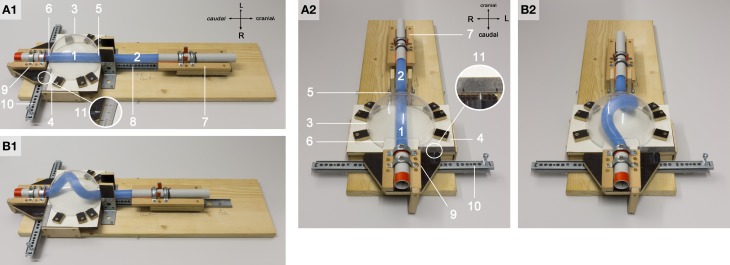
**These photographs document the design of our physical simulation model**. The model is shown in two different views before **(A1,A2)** and during a buckling experiment **(B1,B2)**. Numbers mark the following components: 1, “intra-pericardial” portion of the silicone rod; 2, “extra-pericardial” portion of the silicone rod; 3, hemispherical plastic bowl representing the ventro-lateral walls of the “pericardial” cavity; 4, plane stage representing the dorsal wall of the “pericardial” cavity; 5, opening at the “cranial (arterial)” pole of the “pericardial” cavity; 6, opening at the “caudal (venous)” pole of the “pericardial” cavity; 7, “cranial” sledge carrying the “cranial” end of the rod; 8, ball-bearing rail facilitating exact displacement of the “cranial” sledge along the “cranio-caudal” axis; 9, “caudal” sledge carrying the “caudal” end of the rod; 10, ball-bearing rail facilitating exact displacement of the “caudal” sledge along the “left-right” axis; 11, mm-scale facilitating exact positioning of the center axis of the “caudal” end of the silicone rod along the “left-right” axis (midline corresponds to line 4 on the mm-scale; the position of the center axis of the “caudal” end of the rod is marked by a metal pin fixed on the “caudal” sledge).

The design of our simulation model is shown in Figure 4. All components of the model were installed on a common horizontal plate. A hemispherical bowl of transparent plastic with an inner diameter of 136 mm was chosen to represent the ventro-lateral walls of the “pericardial” cavity. The hemispherical plastic bowl was mounted on a plane stage, which acted as the dorsal wall of the “pericardial” cavity. Two openings were cut in the lateral wall of the hemispherical bowl at opposing sites corresponding to the “cranio-caudal” axis of the model. These openings represented the “cranial” (arterial) and “caudal” (venous) ends of the “pericardial” cavity.

A custom-made flexible rod of solid silicone rubber, 480 mm long with a diameter of 23 mm, was used as “growing heart tube model” for the buckling tests. This solid silicone rod was made from a commercially available two-component system (Thiesasil 17®, Thie & Söhne, Germany; component A: polysiloxanes + fillers, component B: curing additives; mixing ratio A/B = 10:1). It had a rubber hardness of 17 (Shore A scale) and, therefore, can be roughly classified as a soft object. Compared with embryonic heart loops, however, it was a relatively stiff object. Thus, the material properties of our heart-tube model did not correspond to those of real embryonic heart loops. A solid silicone rubber of Shore A hardness 17 was chosen as the best material for our heart tube model since its mechanical properties facilitated (i) the highest possible deformability combined with (ii) full reversibility of buckling-deformations. These mechanical properties minimized the risk of generating falsifying memory effects at repeated buckling tests. We should also note that the silicone rod had no lumen. This means that our model neglected the tubular structure of the embryonic heart loop. We think that this fact does not significantly alter the validity of our simulation model since the early embryonic heart is a thick-walled blood vessel that has only a slim, slit-shaped lumen, which is completely closed during contraction of its myocardial wall.

The straight silicone rod was positioned along the central “cranio-caudal” axis of the model. In the straight state, only a ~136 mm long end of the rod was inside of the “pericardial” cavity while the rest (~344 mm) was outside of the cavity. Thus, we distinguished two portions of the rod: (i) an “intra-pericardial” portion, representing the embryonic heart tube; and (ii) an “extra-pericardial” portion, representing the heart fields from which new material will be added to the heart. The “cranial” end of the rod was mechanically fixed to a sledge, which was anchored on a straight, ball-bearing rail oriented along the “body” midline of the model. This facilitated exact displacements of the “cranial” end of the rod along the “cranio-caudal” axis. The “caudal” end of the rod was mechanically fixed to a sledge, which was anchored on a straight, ball-bearing rail oriented perpendicular to the “cranio-caudal” axis of the model. This facilitated exact displacements of the “caudal” pole of the rod along the “left-right” axis. At the pre-looping state (straight heart tube), the length-to-diameter ratio of our heart tube model was 5.91 (136/23 mm). This value is higher than the corresponding values found in vertebrate embryos (e.g., chick 3.94, human 2.63, Xenopus 1.85). This means that our heart tube model was slimmer than real embryonic heart loops. The slim design was chosen since our model was stiffer than embryonic heart loop (see above). This reduced the compressive forces needed for the generation of buckling-deformations.

For the simulation of the cranio-caudal growth of the heart tube, the sledge carrying the “cranial” end of the rod was gradually displaced along the rail toward the “caudal” end of the model (Figures 4B1,B2). Bilateral symmetric (midline) or asymmetric (left- or right-sided) positions of the “venous heart pole” were accomplished by displacements of the sledge carrying the “caudal” end of the rod. In order to avoid falsifying memory effects, buckling tests with asymmetric (left- or right-sided) positioning of the “venous heart pole” were carried out in alternating fashion.

We should note here that, in our looping model, the cranio-caudal growth of the heart tube was simulated by the addition of new material only to its “cranial” end so that the axial compressive force was also applied only to the “cranial” end of the silicone rod. This differs from the *in vivo* situation where the embryonic heart loop becomes elongated by addition of new material to its cranial as well as caudal ends (van den Berg et al., 2009; Kelly, 2012). From a physical viewpoint, however, it makes no difference whether the axial compressive load is applied only to one end or to both ends of the rod. Confining growth simulation to the “cranial” pole of our heart loop model simplified the construction of its “caudal” pole, which now had to facilitate only the simulation of left- and right-ward displacements of the venous heart pole. Thus, the present design of our model was chosen since it made the handling of our model as simple as possible.

Frictional forces have profound influences on buckling configurations (Tan and Forsman, 1995). In order to reduce frictional forces between the silicone rod and the walls of the “pericardial” cavity, the surface of the silicone rod was coated with a polymethylmethacrylate powder (Paladon® 65; Heraeus Kulzer GmbH, Germany; particle size 40 – 160 μm).

We expected that sinusoidal buckling (planar bending) of the “intra-pericardial” portion of the silicone rod would not occur along a favored plane. This differs from cardiac looping where the bending process is consistently directed toward the ventral body wall. In order to direct the sinusoidal buckling of the “intra-pericardial” portion of the silicone rod toward the ventral wall of the “pericardial” cavity, the center axes of its “cranial” and “caudal” ends were slightly tilted toward the mid-ventral aspect of the “pericardial” cavity. These slight deviations of the center axis of the silicone rod from a linear “cranio-caudal” axis correspond to the situation found in the straight heart tube of human embryos with 7 somites (see Figure 4 in De Vries and Saunders, 1962).

All experiments were conducted at a constant room temperature of 25°C. During the buckling experiments, photos were taken at standard views (frontal, right lateral, left lateral) to record the buckling configurations.

## Results

### Experiment 1

The first set of buckling tests was conducted to clarify whether growth-induced buckling of a straight elastic rod (representing the embryonic heart tube) in a narrow hemispherical cavity (representing the embryonic pericardial cavity) can generate the same sequence of form changes as observed in the looping embryonic heart. These tests were conducted under bilaterally symmetric conditions (center axis of the two ends of the rod in midline position).

**Figure 5:**
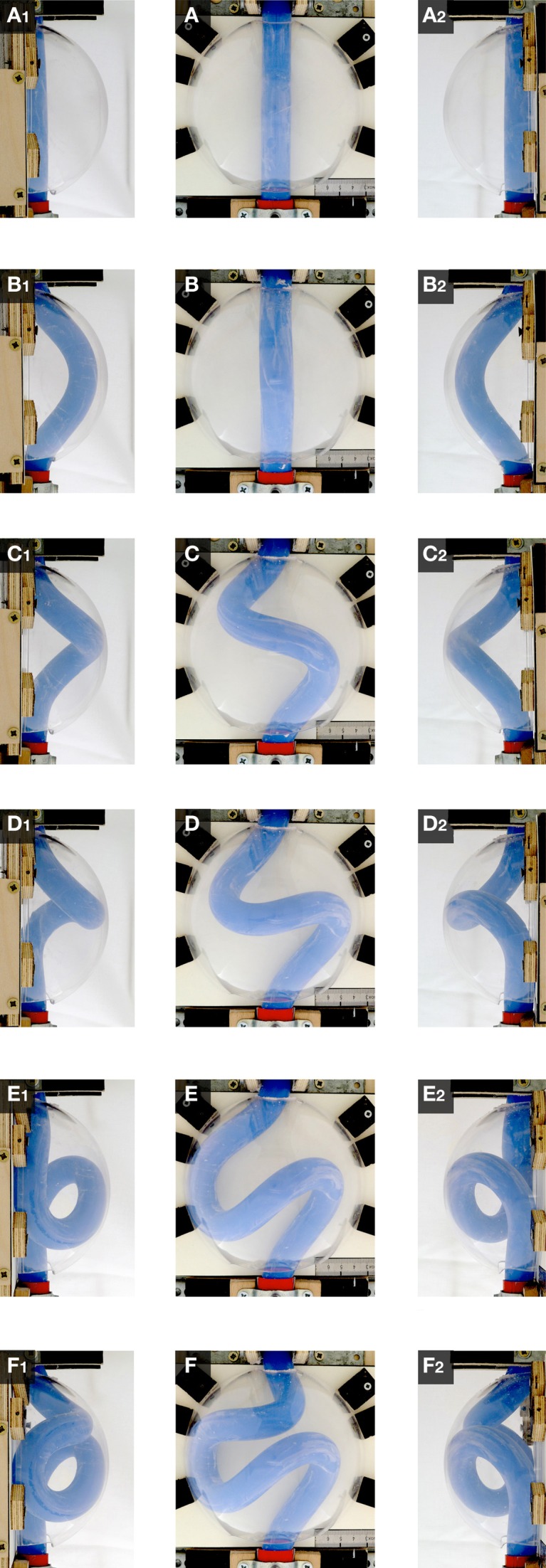
**These photographs taken at frontal (A–F), right-lateral (A1–F1), and left-lateral views (A2–F2), document the shape changes of the continuously elongating “intra-pericardial” portion of the silicone rod during the first buckling experiment**. **(A,A1,A2)** Straight rod. **(B,B1,B2)** Sinusoidal buckling along the mid-sagittal plane. **(C,C1,C2–E,E1,E2)** Simple helical buckling (here: left-handed helix). **(F,F1,F2)** Complex helical buckling = deformation of a single-handed helix into a two-handed helix (here: left-handed helix → LR-helix). Compare the buckling configurations with the idealized geometrical heart loop configurations shown in Figure 1.

**Figure 6:**
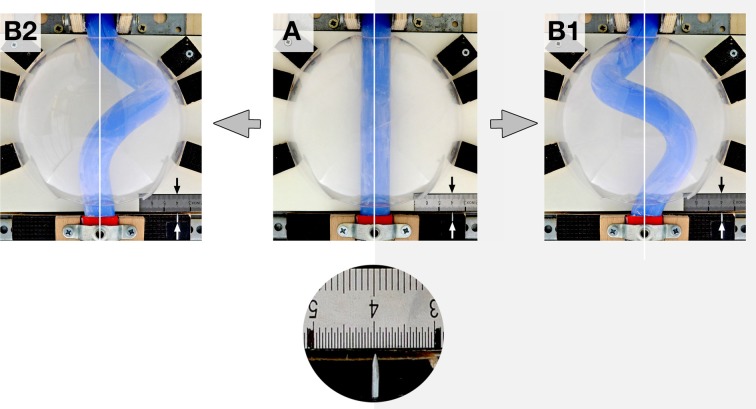
**These photographs taken at frontal views illustrate the fact that, under bilaterally symmetric conditions, helical buckling generated left- as well as right-handed helices**. **(A)** “Intra-pericardial” portion of the silicone rod before starting of the buckling test. Note the midline position of the center axis of the straight silicone rod [midline marked by white lines and line 4 on the mm-scale (black arrow); position of the center axis of the “caudal” end is indicated by a metal pin pointing to the mm-scale (white arrow)]. **(B1,B2)** “Intra-pericardial” portion of the silicone rod during helical buckling. Photographs show a left-handed helix **(B1)** and a right-handed helix **(B2)**. Note that the center axes of the “cranial” and “caudal” ends of the silicone rod are still in their original midline positions.

**Figure 7:**
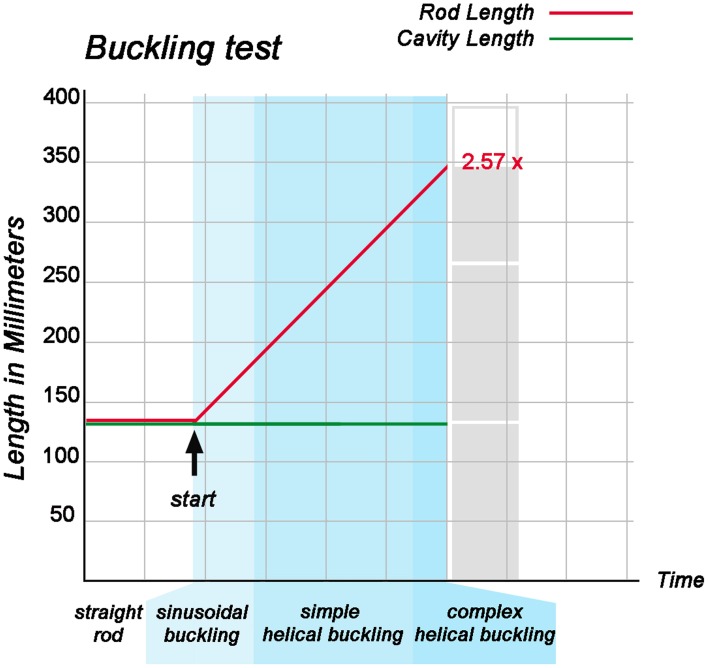
**This graph illustrates the relation between the rate of elongation of the “intra-pericardial” portion of the silicone rod and its form changes during the first buckling experiment**. During the whole experiment, the “cranio-caudal” length of the “pericardial” cavity remained constant, while the “intra-pericardial” portion of the silicone rod underwent considerable elongation. At the start of the buckling test, the “intra-pericardial” portion of the silicone rod was straight and had the same length as the “pericardial” cavity (136 mm; length-to-diameter ratio for the rod = 5.9). Lengthening of the “intra-pericardial” portion of the rod led to sinusoidal buckling. The switch from sinusoidal to simple helical buckling occurred at 183 mm length (length-to-diameter ratio = 7.96), while the switch from simple to complex helical buckling occurred at 327 mm length (length-to-diameter ratio = 14.22). At the end of the buckling test, the length of the “intra-pericardial” portion of the rod was 350 mm (length-to-diameter ratio = 15.22), which is 2.57 times longer than the “pericardial” cavity.

When the “cranial” end of the rod was gradually displaced toward its fixed “caudal” end, the rod became subjected to an increasing axial load and the originally “extra-pericardial” portion of the rod was gradually displaced into the “pericardial” cavity. As a consequence, the “intra-pericardial” portion of the rod increased in length and was gradually bent (Figures 5A,B). Due to the fact that the center axes of the “cranial” and “caudal” ends of the “intra-pericardial portion” of the rod were slightly tilted toward the mid-ventral aspect of the “pericardial” cavity (see Materials and methods), the bending process was consistently directed toward the ventral wall of the “pericardial” cavity and was confined to a single plane corresponding to the mid-sagittal plane of the vertebrate body. When the bending rod contacted the ventral wall of the “pericardial” cavity, the two-dimensional bending switched to three-dimensional coiling and the rod acquired the shape of a helix with a single winding (Figure 5C). At repeated tests (*n* = 50), left- and right-handed helices occurred almost at the same ratio (Table 1; Figure 6) indicating that, under bilaterally symmetric conditions, there was no preference for the generation of a particular helix enantiomorph. Further increase in length of the “intra-pericardial” portion of the rod was accompanied by gradual decrease of the pitch of the helix (Figures 5D,E) until the rod finally acquired the configuration of a two-handed helix (Figure 5F). Deformation of “single-handed” helices into two-handed helices occurred in such a way that a left-handed helix consistently acquired the shape of a LR-handed helix (Figures 5D–F), while a right-handed helix acquired the shape of a RL-handed helix (not shown). When summarizing the results of these buckling tests, we can state that the buckling process observed in our physical model is characterized by three subsequent steps that may be named: (i) sinusoidal buckling, (ii) simple helical buckling, and (iii) complex helical buckling. Starting with an initial length of 136 mm (straight rod), the continuously elongating “intra-pericardial” portion of the rod switch from sinusoidal to helical buckling at 183 mm length, while the switch from simple to complex helical buckling occurred at 327 mm length (Figure 7). At the end of the buckling experiment, the length of the “intra-pericardial” portion of the rod was 2.57 times longer than the length of the “pericardial” cavity (Figure 7).

**Table 1:** **Relations between pre-buckling positions of the “caudal” end of the silicone rod and the directionality of helical buckling**.

	**Left-handed helix *D-loop* enantiomorph**	**Right-handed helix *L-loop* enantiomorph**
**MIDLINE POSITION**		
*n* = 50	28 (56%)	22 (44%)
**LEFTWARD DISPLACEMENT**		
1 mm (*n* = 50)	47 (94%)	3 (6%)
2 mm (*n* = 50)	50 (100%)	–
5 mm (*n* = 50)	50 (100%)	–
10 mm (*n* = 50)	50 (100%)	–
**RIGHTWARD DISPLACEMENT**		
1 mm (*n* = 50)	–	50 (100%)
2 mm (*n* = 50)	–	50 (100%)
5 mm (*n* = 50)	–	50 (100%)
10 mm (*n* = 50)	–	50 (100%)

### Experiment 2

**Figure 8:**
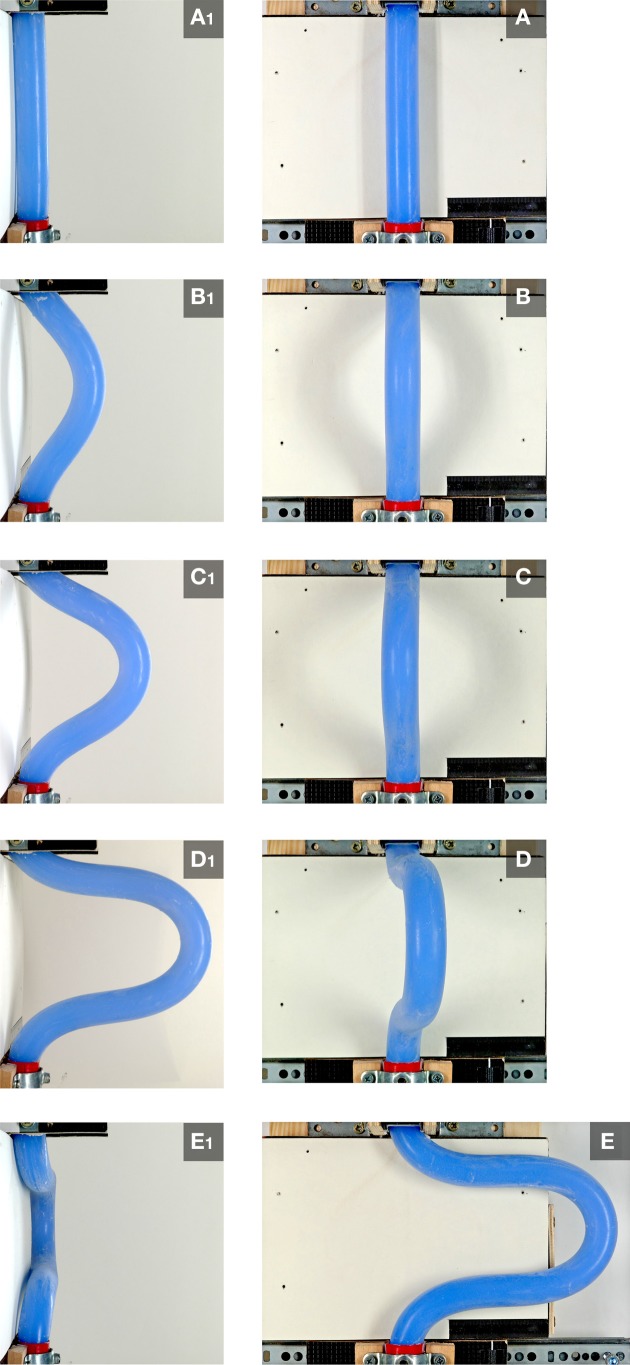
**These photographs, taken at frontal (A–E) and right-lateral views (A1–E1), document the buckling behavior of the continuously elongating “intra-pericardial” portion of the silicone rod in the absence of the hemispherical plastic bowl**. Under this condition, sinusoidal buckling did not switch to helical buckling. Sinusoidal buckling continued until the configuration became unstable under the rods' own weight and the curved loop flapped either to the left **(E,E1)** or right side.

In order to clarify the role of the mechanical boundaries of the “pericardial” cavity in the buckling process, the tests were repeated in the absence of the hemispherical plastic bowl. Under this condition, sinusoidal buckling did not switch to simple or complex helical buckling. The continuously elongating rod was progressively bent along the mid-sagittal plane until the configuration became unstable under the rods' own weight and the curved loop flapped either to the left or right side (Figure 8).

### Experiment 3

**Figure 9:**
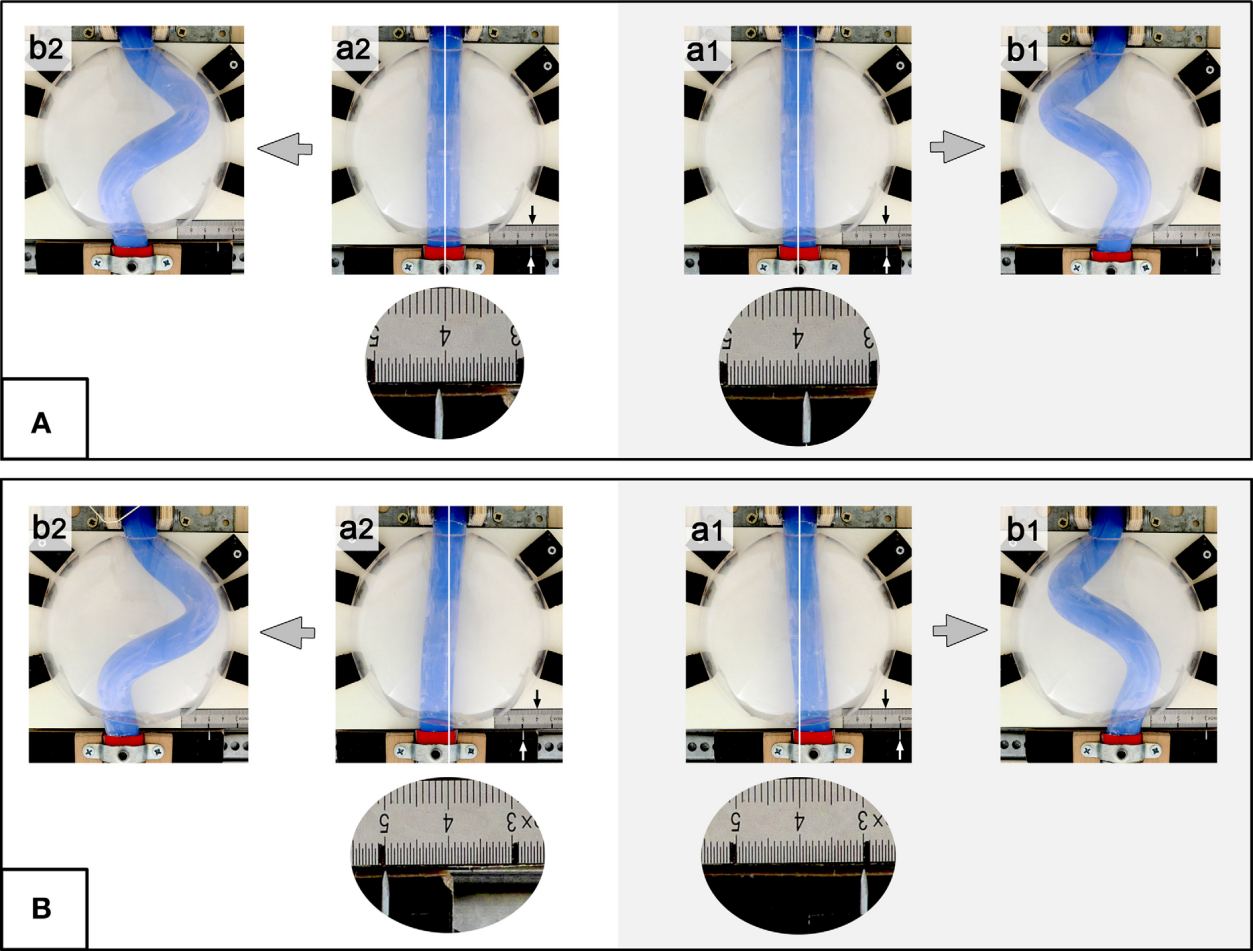
**These photographs taken at frontal views show examples of buckling tests conducted under asymmetric conditions (experiment 3: left- or rightward displacement of the “caudal” end of the silicone rod)**. **(A)** 1 mm lateral displacement before running of the buckling experiment. **(B)** 10 mm lateral displacement before starting the buckling experiment. Note that pre-buckling displacement toward the left **(a1)** produced left-handed helices **(b1)**, while rightward displacement **(a2)** produced right-handed helices **(b2)**. The “body” midline is marked by white line and line 4 on the mm-scale (black arrow). The position of the center axis of the “caudal” end is indicated by a metal pin pointing to the mm-scale (white arrow). Round and oval photographs are higher magnification views of the mm-scale.

The determination of the handedness of the embryonic heart loop has been attributed to pre-existing left-right asymmetries (Stalsberg, 1970), such as subtle leftward displacement of the caudal end of the straight heart tube (von Baer, 1828; Schulte, 1916; Biben and Harvey, 1997). Thus, in the third experiment, the buckling tests were conducted under bilaterally asymmetric conditions, caused by left- or rightward displacement of the “caudal” end of the silicone rod at the pre-buckling state. Under these conditions, the buckling process was indeed directed toward the generation of only one of two possible helix enantiomorphs (Table 1; Figure 9). Leftward displacement directed the helical buckling process toward the generation of a left-handed helix, while rightward displacement directed the buckling process toward the generation of a right-handed helix. Even 1 mm displacement toward the left or right side, which was less than 5% of the diameter of the rod, was sufficient to direct the buckling process toward an almost 100% generation of left- or right-handed helices, respectively (Table 1).

### Experiment 4

**Figure 10:**
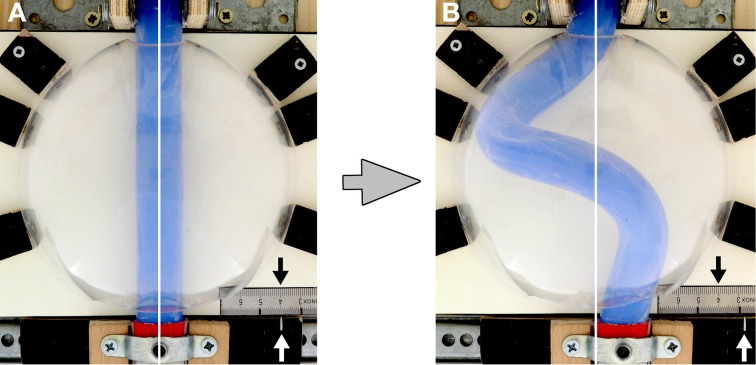
**These photographs taken at frontal views depict the leftward displacement of the “caudal” end of the silicone rod caused by left-handed helical buckling. (A)** Straight rod before starting of the buckling test. Note the midline position of the center axis of the silicone rod. **(B)** Silicone rod during helical buckling. Note the leftward displacement of the “caudal” end of the rod.

In the above-mentioned three experiments, the “caudal” end of the rod was mechanically fixed in its initial midline (Experiments 1 + 2), left-sided, or right-sided positions (Experiment 3) during the whole buckling test. During D-looping of the embryonic vertebrate heart, however, the caudal end of the straight heart tube (future atrio-ventricular canal) does not remain in its initial position but moves continuously further to the left (Murray, 1919; De Vries and Saunders, 1962; Biben and Harvey, 1997). We wanted to know whether this movement could be a consequence of the buckling process. Thus, in the final experiments, the “caudal” end of the rod was allowed to move along the left-right axis during the buckling tests. We found that, under these conditions, the emergence of a left-handed helix was accompanied by leftward displacement of its “caudal” end (Figure 10), while the emergence of a right-handed helix led to rightward displacement of its “caudal” end (not shown).

## Discussion

During the past 200 years, research on cardiac looping has focused on the answering of two main questions: (i) what factors drive the normal positional and morphological changes of the looping embryonic heart tube; and (ii) what factors determine its handedness? The buckling experiments conducted in the present study may help to answer both questions since they may contribute to the identification of mechanical factors involved in cardiac looping. With respect to the first question, we have found that growth-induced buckling of a straight elastic rod within the confined space of a hemispherical cavity can generate a sequence of form changes corresponding to the sequence of form changes characterizing the looping morphogenesis of the tubular heart of higher vertebrate embryos (Figure 5). Both processes start with bending of the initially straight rod/tube along a single plane (Figure 5B). When the bending rod/tube contacts the wall of the confining cavity, the two-dimensional bending converts to three-dimensional coiling and the curved rod/tube acquires the shape of a “single-handed” helix (Figures 5C–E) and finally the configuration of a two-handed-helix (Figure 5F). In our buckling experiment, the length of the two-handed helix (representing the “S-shaped” heart) was 2.57 times longer than the length of the “pericardial” cavity (Figure 7). This value fits nicely to the corresponding value measured in Axolotl embryos (× 2.64; Figure 2A) but is smaller than the value measured in chick embryos (× 3.37; Figure 2B) and is somewhat larger than the value reported for human embryos (× 2.0; Davis, 1927). These differences may be explained by differences in shapes and/or relative sizes of the heart tube and pericardial cavity (e.g., length-to-diameter ratio of the heart tube). We have, furthermore, found that the emergence of simple and complex helical configurations depends on the presence of mechanical boundaries provided by the walls of a hemispherical cavity (Figure 8). All these observations are in good agreement with the growth-induced buckling hypothesis of cardiac looping mechanics, which attributes the form changes of the looping embryonic heart mainly to compressive forces resulting from growth differences between the heart and the pericardial cavity. With respect to the second question, our simulation experiments have shown that, under bilaterally symmetric conditions, helical buckling generates left- and right-handed helices in a 1:1 ratio, while even subtle left- or rightward displacements of the lower end of the elastic rod at the pre-buckling state are sufficient to direct the buckling process toward the generation of only left- or right-handed helices, respectively (Table 1, Figures 6, 9). These observations are in good agreement with the idea that a subtle leftward displacement of the caudal end of the heart tube at pre-looping stages may be a decisive event in determining the D-loop enantiomorph (von Baer, 1828; Schulte, 1916; Biben and Harvey, 1997). Thus, we can summarize that our physical simulation model demonstrates, for the first time, the physical plausibility of the growth-induced buckling hypothesis of cardiac looping mechanics. It, furthermore, demonstrates that the idea of a handedness-determining role of an asymmetric positioning of the venous heart pole (von Baer, 1828; Schulte, 1916; Biben and Harvey, 1997) is physically plausible in the context of helical buckling morphogenesis. Therefore, we think that we should now do the second step and check the buckling hypotheses for its biological plausibility.

### Remarks on the role of unequal growth in looping morphogenesis

According to the growth-induced buckling hypothesis, the looping embryonic heart tube is deformed mainly by two mechanical forces: (i) an axial compressive load, resulting from a difference in cranio-caudal growth between the heart tube and the embryonic pericardial cavity (Figure 2); and (ii) a compressive load resulting from the passive mechanical restriction of the sinusoidal bending of the heart tube by the ventro-lateral walls of the embryonic pericardial cavity. The strengths of both forces should depend on the growth behaviors of the heart tube and of the pericardial cavity. For example, if the rate of lengthening of the embryonic heart tube would be reduced to a level corresponding to the rate of cranio-caudal lengthening of the pericardial cavity, the expected value for the axial compressive load is zero and the expected heart configuration should be a straight tube. The qualitatively same phenotype (straight heart tube) should occur if the rate of cranio-caudal lengthening of the pericardial cavity would be elevated to a level corresponding to the rate of lengthening of the embryonic heart tube. Excessive growth of the heart within a normal-sized pericardial cavity or normal cardiac growth within a growth-deficient pericardial cavity, on the other hand, are expected to cause abnormally high values for the axial compressive load with the final results of highly convoluted heart loops.

Almost all of the above-mentioned hypothetical situations have already been documented in animal models showing abnormalities in cardiac looping. Pictures of embryos reported to develop non-looping (straight tube) or incomplete looping phenotypes show either an abnormally short heart tube within a normal-sized pericardial cavity (e.g., Figure 1 in Yelbuz et al., 2002; Figure 5 in Brade et al., 2007; Figure 2 in Klaus et al., 2007; Figure 1 in Niu et al., 2008; Figure 5 in Choudhry and Trede, 2013), or an almost normal-sized heart tube within an abnormally enlarged pericardial cavity (e.g., Figure 1 in Garrity et al., 2002; Figure 5 in Breckenridge et al., 2001; Figure 3 in Ribeiro et al., 2007; Figure 2 in Doherty et al., 2010). Pictures of embryos reported to have excessive looping phenotypes, on the other hand, show an abnormally long heart tube within a normal-sized pericardial cavity (Figure 3 in Risebro et al., 2006). These data suggest that a significant number of cardiac looping anomalies found in diverse animal models, especially in models with impaired development of the heart-forming fields, may not primarily result from disturbances in specific form-generating or form-regulating processes but rather may result from altered growth of the heart and/or the pericardial cavity. Thus, in agreement with the growth-induced buckling hypothesis, unequal growth of the heart and its confining pericardial cavity seems to be an important determinant of cardiac looping morphogenesis not only in our present simulation model but also in the biological reality.

### Remarks on the mechanics of ventral bending

Ventral bending is the first prominent shape change of the looping embryonic heart tube. The growth-induced buckling hypothesis regards such bending as a deforming process (sinusoidal buckling) resulting from axial compression of the tubular heart. Our present simulation model confirmed the physical plausibility of this concept. It is a well-known fact, however, that tests on biological models, such as *in vitro* cultures of isolated embryonic heart tubes, seem to have disproven the biological plausibility of the buckling concept. If ventral bending of the embryonic heart tube would be exclusively a process of sinusoidal buckling, we should expect that a straight heart tube will not be able to bend when it is removed from the pericardial cavity and cultured as isolated specimen in an *in vitro* culture system. In contrast to this expectation, however, it has been shown that isolated embryonic heart tubes have an intrinsic capacity for ventral bending (Ekman, 1925; Bacon, 1945; Butler, 1952; Manning and McLachlan, 1990; Flynn et al., 1991). Moreover, some experimental data suggest that the intrinsic bending of embryonic heart loops may be based on active myocardial cell shape changes driven by actin polymerization (Latacha et al., 2005; Rémond et al., 2006; Taber, 2006). In view of these findings, it seems unlikely that the ventral bending of the embryonic heart tube results mainly from growth-induced axial compression. In view of the observations discussed in the preceding paragraph, however, it also seems unlikely that the ventral bending of the embryonic heart tube depends exclusively on intrinsic bending mechanisms in the intact embryo. We speculate that, in biological reality, active myocardial cell shape changes might play a leading role in ventral bending of the heart tube while the longitudinal growth behaviors of the embryonic heart and pericardial cavity might play permitting rather than active roles in this process. We should note, however, that not all available data seem to support the idea of an intrinsic, cell shape-driven bending mechanism. Hill and Lemanski (1979) could not observe a progression of heart looping in *in vitro* cultures of amphibian embryonic heart loops, and data from colchicine-treated chick embryos suggest that the cell shape changes observed during ventral bending are merely the result and not the cause of the bending process (Icardo and Ojeda, 1984).

### Remarks on the mechanics of simple and complex helical coiling

*In vitro* looping of isolated embryonic heart tubes is a planar bending process that does not generate simple or complex helical configurations (Flynn et al., 1991; Latacha et al., 2005; Rémond et al., 2006; Ramasubramanian et al., 2013). Thus, in contrast to ventral bending, the transformation of a curved tube into a helical loop is mainly attributed to forces outside of the developing heart (Taber, 2006; Taber et al., 2010). It is tempting to speculate that such extrinsic forces might act in a bilaterally asymmetric fashion to generate a bilaterally asymmetric object such as a helically coiled heart loop. Our present simulation experiments, however, have shown that helical configurations can evolve in a buckling process that runs under bilaterally symmetric conditions, whereby the initial symmetry finds its expression in a statistically random generation of the two possible enantiomorphs. We, therefore, speculate that, in vertebrate embryos, the emergence of helical heart loop configurations may not necessarily depend on the presence of structural or functional asymmetries suspected to drive the helical deformation of the curved heart loop. We suspect that such asymmetries might rather play a prominent role in the determination of heart loop handedness. This idea is, indeed, supported by experimental data from chick and zebrafish embryos, which show that the artificial creation of molecular and morphological symmetry does not prevent the looping morphogenesis of the embryonic heart tube but only leads to a random emergence of the heart loop enantiomorphs (Breckenridge et al., 2001; Kidokoro et al., 2008). We, therefore, discuss the mechanics of helical looping and of the determination of heart loop handedness in separate paragraphs.

Our simulation model has shown that, in a buckling process, the emergence of simple and complex helical loop configurations depends on the presence of mechanical boundaries that passively restrict the sinusoidal bending of a continuously elongating rod or tube. Thus, if the helical deformations of the looping embryonic heart would result from a buckling process, we should expect that a curved heart tube shouldn't be able to acquire a helical shape (i) when it is removed from the pericardial cavity and cultured *in vitro* without spatial restrictions; or (ii) when it develops in a pericardial cavity whose ventro-lateral walls have been removed before or during the initial phase of cardiac looping.

Experimental data from chick embryos have shown (i) that *in vitro* looping of isolated heart tubes does not produce helical heart loops (see above); and (ii) that the removal of the ventral wall of the primary pericardial cavity near the onset of looping prevents the torsion (rotation) which normally deforms the bending heart tube into a helically wound heart loop (Voronov and Taber, 2002; Voronov et al., 2004). Both observations seem to be in accord with the above-mentioned two expectations and, therefore, may be regarded as supporting the growth-induced buckling hypothesis. We should note, however, that other experimental data have shown that the torsion-preventing effect of the removal of the ventral pericardial wall is only of temporary nature and that, in the absence of compressive load exerted by the ventral pericardial wall, heart loop torsion is fully restored by a mechanism that normally does not contribute to dextral-looping of the embryonic chick heart: namely cytoskeletal contractions of the myocardium (Nerurkar et al., 2006). Thus, the currently available experimental data do not provide a clear picture of the morpho-mechanics of simple and complex helical coiling of the embryonic heart loop. On the one hand, we have data suggesting that these form changes normally may evolve in consequence of compressive forces resulting from unequal growth of the heart and pericardial cavity (Voronov and Taber, 2002; Voronov et al., 2004). On the other hand, however, we have data suggesting that the embryonic heart tube may have a latent intrinsic tendency for helical coiling which may act as a regulatory morphogenetic mechanism in the case of absence of the mechanical boundaries formed by the ventral pericardial wall (Nerurkar et al., 2006).

### Remarks on the mechanics of determination of cardiac handedness

The problem of determination of cardiac handedness has fascinated biologists since the nineteenth century. Thus, during the past 200 years, various biophysical mechanisms have been proposed to direct cardiac looping toward the consistent generation of the D-loop enantiomorph. Most of these morpho-mechanical concepts have been disproven by experimental data (Taber, 2006) so that, at the present time, only two well-founded concepts have survived. The first concept attributes the determination of dextra-looping to the asymmetric distribution of mechanical forces along the veins connected to the caudal pole of the straight heart tube. The “stronger” left vein is said to displace the heart slightly toward the right (Taber, 2006; Taber et al., 2010). The second concept attributes the determination of the direction of cardiac looping to bilaterally asymmetric patterns of cell proliferation within the dorsal mesocardium and the foregut endoderm (Linask et al., 2005; Linask and Vanauker, 2007). In our present simulation model we have tested, for the first time, the physical feasibility of a third concept, which attributes the determination of the D-loop enantiomorph to a subtle leftward displacement of the venous heart pole shortly before the onset of looping. The Baltic-German embryologist Karl Ernst von Baer (*1792–1876†) might be the first who recognized the potentially handedness-determining role of this pre-looping asymmetry. He speculated that the leftward displacement of the venous pole might cause an asymmetric routing of the intracardiac blood flow from the left to the right which, in turn, might lead to rightward looping of the developing ventricles (von Baer, 1828). Despite the fact that several researchers noted that such a hemodynamic mechanism conflicted with the fact that cardiac looping started long before the onset of blood flow, von Baer's idea survived for a long time and was still found in some articles dealing with early cardiogenesis at the beginning of the twentieth century (e.g., Schulte, 1916). During the following eight decades, the leftward displacement of the straight heart tube was described in a few papers (Murray, 1919; De Vries and Saunders, 1962) but the idea of a handedness-determining role of this pre-looping asymmetry seems to have been forgotten up to the mid-1990ies when this idea was revived by Biben and Harvey (1997). The latter authors speculated that this early asymmetry “*… may be a decisive event in determining of heart situs*” but, unfortunately, did not present a concept of how this asymmetry may mechanically act in the determination of heart loop handedness. Our present simulation model demonstrates, for the first time, that the idea of a handedness-determining role of an asymmetric positioning of the venous heart pole is physically plausible in the context of helical buckling morphogenesis. Future studies are needed to clarify the biological feasibility of this concept as well as to clarify the relation between the leftward displacement of the pre-looping heart of mammalian embryos and the phenomenon of leftward jogging of the pre-looping heart of zebrafish embryos (Chen et al., 1997; Chin et al., 2000; Khodiyar et al., 2014). Based on the data from our simulation model, we can make predictions, which can be tested in future studies on animal models. In mouse models with non-biased looping (1:1 ratio of D- and L-loop), for example, we would expect to find one of three alternative scenarios at the pre-looping state: (i) a 1:1 ratio of left- and rightward displacements of the venous heart pole; (ii) absence of any lateral displacement of the venous pole (symmetric midline position); or (iii) a 1:2:1 ratio of left-sided, midline, and right-sided positions of the venous heart pole. In mouse models with L-loop bias, on the other hand, we should expect to find a corresponding bias of rightward displacement of the venous heart pole at the pre-looping state. Examples of such rare animal models are (i) mice homozygous for a mutation in the *inversin* gene, which show an almost 100% L-loop bias (Yokoyama et al., 1993); and (ii) mice with a loss of function allele of the *Dnaic1* gene, which show a 60% L-loop bias (Francis et al., 2012).

A handedness-determining role of asymmetric positioning of the venous heart pole may also explain a puzzling phenomenon observed in a developmental anomaly named *cardia bifida*. This anomaly is characterized by the presence of two separate hearts, from which one lies to the left and the other to the right of the body midline. Cardia bifida is an extremely rare finding in post-natal animals and humans (Panum, 1859; Aiello et al., 1987; Aiello and Xavier-Neto, 2006). It is well known to embryologists, however, since it can be produced by microsurgical or genetic defects preventing the merging of the left and right heart-forming fields (Gräper, 1907; Copenhaver, 1926; DeHaan, 1959; Zwirner and Kuhlo, 1964; Lepori, 1967; Nadal-Ginard and Paz Garcia, 1972; Li et al., 2004). In experimental embryos with cardia bifida, the left hemi-heart was reported to acquire almost always the D-loop enantiomorph, while the right hemi-heart acquired the L-loop enantiomorph (Ekman, 1925; Copenhaver, 1926; Zwirner and Kuhlo, 1964; Nadal-Ginard and Paz Garcia, 1972). This phenomenon led to the idea, that each heart-forming field may harbor its own intrinsic enantiomorphic tendency (Weiss, 1930; Zwirner and Kuhlo, 1964), which may become apparent only in the case of failure of merging of the heart-forming fields. In the case of merging, however, the enantiomorphic tendency of the right heart-forming field normally may remain latent since it may be dominated by the enantiomorphic tendency of the left heart-forming field (Weiss, 1930; Zwirner and Kuhlo, 1964). The idea of a morphogenetic dominance of the left heart-forming field seemed to be supported by the observation that, in cases of cardia bifida, the left hemi-heart generally appeared to be of larger size and to have earlier, faster and stronger pulsations compared to the right hemi-heart (Bacon, 1945; DeHaan, 1958, 1959; Zwirner and Kuhlo, 1964; Lepori, 1967). Similar observations were made in cultures of isolated heart-forming fields (Goerttler, 1928; Rawles and Williers, 1934). As tempting as the idea of a left-sided morphogenetic dominance may appear, it has been disproven by a number of experiments (Ekman, 1925; Stöhr, 1925, 1926; Fales, 1946) so that, at the present time, we have no plausible explanation for the pattern of cardiac handedness usually observed in cardia bifida. If one looks on pictures showing the heart situs of amphibian embryos with cardia bifida (e.g., Figure 26 in Copenhaver, 1926), one will note that the arterial poles of the two hemi-hearts are positioned close to the body midline while their venous poles are positioned distant from the body midline. Thus, in the right hemi-heart, which usually develops the L-loop enantiomorph, the venous pole lies to the right of the arterial pole, while in the left hemi-heart, which usually develops the D-loop enantiomorph, the venous pole lies to the left of the arterial pole. In view of the results of our present buckling experiments, it is tempting to speculate that such positional relationships between the fixed ends of the developing hemi-hearts are responsible for the emergence of the pattern of cardiac handedness usually observed in cardia bifida.

### Remarks on the mechanical stability of post-buckling configurations

The post-buckling configurations of an originally straight elastic rod/tube are not stable. If the compressive loads are removed, the rod/tube tends to return to its original shape in consequence of its elasticity. A corresponding behavior was observed in chick embryos when the ventral wall of the primary pericardial cavity was removed during the initial phase of dextral-looping (HH-stage 11). In this case, the heart loop lost its already acquired helical shape and returned to a sinusoidal configuration (Voronov and Taber, 2002; Voronov et al., 2004). However, it is a well-known fact to embryologists that, if the ventral pericardial wall is removed at advanced stages of cardiac looping (e.g., HH-stage 18), the heart loop usually retains its complex helical shape. Thus, if we suspect that the helical deformations of the embryonic heart loop result mainly from growth-induced compressive loads, the latter finding implies the existence of form stabilizing mechanisms within the developing heart. The existence of such mechanisms has been postulated and experimentally confirmed on amphibian embryonic tissues by the Russian biologist Beloussov (Beloussov and Grabovsky, 2006; Kremnyov et al., 2012). Therefore, the mechanical stability of embryonic heart loop configurations at advanced stages of the looping process does not conflict with the growth-induced buckling hypothesis of cardiac looping mechanics.

### Limitations of our simulation model

Every simulation model has its limitations since it focuses only on a clearly defined number of factors suspected to contribute to a given process. Thus, the picture of the looping process provided by our present model may lead to an overrating of the mechanical factors acting in this model. Previous experimental data suggest that cardiac looping may be driven by several independently regulated mechanical factors which generate overlapping effects and may act in a cooperative manner to generate the normal form changes of the looping embryonic heart tube (Stalsberg, 1970; Manasek, 1981). The data from our model do not exclude the possibility that further mechanical factors may play prominent roles in cardiac looping. Among the list of possibly important mechanical factors neglected in our present simulation model are (i) active, cytoskeleton-driven changes in myocardial cell shape which appear to drive the intrinsic bending of the heart tube (see above); (ii) regional differences in mechanical properties (stiffness) of the heart tube which may support the bending caused by active cell-shape changes (Zamir et al., 2003); (iii) regional differences in the proliferation of the ventricular myocardium which may contribute to cardiac looping via expansion (ballooning) of the outer curvature of the ventricular bend (de Boer et al., 2012); (iv) heart loop compression caused by formation of embryonic body (cervical) flexures, which plays an important role in ‘S-looping’ (Männer et al., 1993, 1995; Männer, 2004; Ramasubramanian et al., 2013); (v) bilaterally asymmetric distributions of compressive and tensile stresses along the venous pole of the heart tube which appear to play prominent roles in rightward torsion of the embryonic chick heart (Ramasubramanian et al., 2006; Taber, 2006; Taber et al., 2010); and (vi) bilaterally asymmetric patterns of cell proliferation (L>R) in the dorsal mesocardium and in the foregut endoderm which also may contribute to rightward torsion of the heart loop of chick embryos (Linask et al., 2005; Linask and Vanauker, 2007).

A further limitation of our model is the fact that it is primarily a qualitative model, which uses elements of idealized geometrical configurations and does not include realistic material properties (see Materials and methods). Thus, the morphogenetic behavior observed in our physical model for cardiac looping is an idealized picture that cannot match exactly with the morphogenetic behavior of real embryonic hearts as observed in popular biological “models,” such as zebrafish, frog (*Xenopus laevis*), chick, quail, and mouse. It will be a challenge for the future to construct models facilitating testing of the physical plausibility of the growth-induced buckling hypothesis under more realistic conditions.

We should finally emphasize that our present simulation model has confirmed only the physical plausibility of the growth-induced buckling hypothesis of cardiac looping mechanics. It is not a proof for validity of this hypothesis in real biological systems. Biological objects of the same geometrical shape can be generated by different sets of forces. Because of this phenomenon, named *phenocopy*, we cannot be sure that the forces acting in our model also act in cardiac looping. To illustrate the phenocopy problem, we should note that, recently, one of us (Jörg Männer) has found that the morphogenesis of fastened tendrils of climbing plants is characterized by the same global form changes as the looping morphogenesis of embryonic hearts (Männer, 2013). This finding led to the speculation that cardiac looping and tendrils coiling may be driven by the same morphogenetic mechanism (Männer, 2013). In contrast to our present model, the bending and helical coiling of tendrils do not result from a buckling process but result from a different morphogenetic process named “*intrinsic curvature driven morphogenesis*” (Goriely and Tabor, 1998; McMillen and Goriely, 2002). Future studies are needed to clarify which of the currently discussed hypotheses of cardiac looping mechanics fits best with the biological reality.

### Conflict of interest statement

The authors declare that the research was conducted in the absence of any commercial or financial relationships that could be construed as a potential conflict of interest.
